# What impacts students’ satisfaction the most from Medicine Student Experience Questionnaire in Australia: a validity study

**DOI:** 10.3352/jeehp.2023.20.2

**Published:** 2023-01-18

**Authors:** Pin-Hsiang Huang, Gary Velan, Greg Smith, Melanie Fentoullis, Sean Edward Kennedy, Karen Jane Gibson, Kerry Uebel, Boaz Shulruf

**Affiliations:** 1 Faculty of Medicine & Health, The University of New South Wales, Sydney, Australia; 2 School of Biomedical Sciences, Faculty of Medicine & Health, The University of New South Wales, Sydney, Australia; 3 Department of Medicine, Taipei Veterans General Hospital, Taipei, Taiwan; 4 Centre for Medical and Health Sciences Education, University of Auckland, Auckland, New Zealand; Hallym University, Korea

**Keywords:** Feedback, Medical students, Personal satisfaction, Reproducibility of results, Statistical factor analysis

## Abstract

**Purpose:**

This study evaluated the validity of student feedback derived from Medicine Student Experience Questionnaire (MedSEQ), as well as the predictors of students’ satisfaction in the Medicine program.

**Methods:**

Data from MedSEQ applying to the University of New South Wales Medicine program in 2017, 2019, and 2021 were analyzed. Confirmatory factor analysis (CFA) and Cronbach’s α were used to assess the construct validity and reliability of MedSEQ respectively. Hierarchical multiple linear regressions were used to identify the factors that most impact students’ overall satisfaction with the program.

**Results:**

A total of 1,719 students (34.50%) responded to MedSEQ. CFA showed good fit indices (root mean square error of approximation=0.051; comparative fit index=0.939; chi-square/degrees of freedom=6.429). All factors yielded good (α>0.7) or very good (α>0.8) levels of reliability, except the “online resources” factor, which had acceptable reliability (α=0.687). A multiple linear regression model with only demographic characteristics explained 3.8% of the variance in students’ overall satisfaction, whereas the model adding 8 domains from MedSEQ explained 40%, indicating that 36.2% of the variance was attributable to students’ experience across the 8 domains. Three domains had the strongest impact on overall satisfaction: “being cared for,” “satisfaction with teaching,” and “satisfaction with assessment” (β=0.327, 0.148, 0.148, respectively; all with P<0.001).

**Conclusion:**

MedSEQ has good construct validity and high reliability, reflecting students’ satisfaction with the Medicine program. Key factors impacting students’ satisfaction are the perception of being cared for, quality teaching irrespective of the mode of delivery, and fair assessment tasks which enhance learning.

## Introduction

### Background/rationale

Feedback regarding students’ learning experience is critical for improving teaching and learning efficacy. The University of New South Wales (UNSW) medicine program has established a student evaluation tool, the Medicine Student Experience Questionnaire (MedSEQ), to capture students’ perspectives on their learning experiences in the program. The original questionnaire was developed as part of a redesign of the Medicine program in the early 2000s [1]. The objectives of the evaluation were twofold: understanding aspects of students’ learning experiences and utilizing students’ feedback for program improvement and further development. Students in each year of the 6-year undergraduate medicine program are invited to complete MedSEQ every 2 years. In 2017, the MedSEQ questionnaire was revised, based on best current practice [2,3]. Since that revision, students responded to the updated version of MedSEQ 3 times, in 2017, 2019, and 2021. It is important to note that the 2021 MedSEQ was heavily impacted by the coronavirus disease 2019 (COVID-19) pandemic, during which major disruptions to teaching, learning and assessment occurred. This resulted in many students spending long periods studying remotely online either domestically or overseas, limiting their exposure to clinical learning experiences.

### Objectives

This study aimed to evaluate the validity of student feedback derived from MedSEQ, and to identify the predictors of students’ satisfaction with the Medicine program.

## Methods

### Ethics statement

Ethics approval number HC210374 was granted by the University of New South Wales review panels in 2021 which enabled analysis of this retrospective study. The requirement for informed consent from an individual participant was omitted because of the retrospective design of this study.

### Study design

A retrospective cross-sectional descriptive study was conducted to evaluate the validity of MedSEQ and to identify the predictors of students’ satisfaction in the medicine program via questionnaire responses from 3 cohorts.

### Setting

The UNSW Medicine program is an undergraduate program, 6 years in duration, with approximately 280 students per year. Teaching is provided at the central university campus and 9 clinical campuses, including 4 rural sites. Students in each year of the medicine program are invited to complete MedSEQ every 2 years. An email invitation to respond to MedSEQ (Supplement 1) was sent to all students enrolled in the UNSW medicine program in October 2017, October 2019, and October 2021. This online survey was open to students throughout those months.

### Participants

All UNSW medical students were invited to respond anonymously to MedSEQ in October 2017, October 2019, and October 2021. There were no exclusion criteria for participation. Medical students were incentivized to participate by a random draw for a small monetary prize AUD250) awarded to 3 students (one from each phase of the program).

### Variables

Variables included cohort year, study year, students’ campus location (metropolitan, rural, or overseas), gender, and 23 additional MedSEQ items regarding students’ learning experiences. The dependent variable was the last question in MedSEQ, which enabled students to rate their overall level of satisfaction with their experience in the medicine program.

### Data sources/measurement

MedSEQ was developed for UNSW medicine program in the early 2000s with acceptable reliability (Cronbach’s α between 0.63 and 0.80) [1]. MedSEQ was revised in 2017 (Supplement 1) based on current guidelines regarding scale development [2,3], in which by using exploratory factor analysis, 8 domains were identified (satisfaction with teaching; satisfaction with assessment; support by staff; learning opportunities; clinical resources; online resources; cultural education; being cared for). The questionnaire was distributed biennially to students enrolled in the UNSW medicine program. Response data are available at Dataset 1.

### Bias

Response bias might have existed due to the nature of survey study.

### Study size

Although there are no clear guidelines regarding sample size calculation for confirmatory factor analysis (CFA), a factor analysis sample size of 50, 100, 200, 300, 500, and 1,000 are generally regarded as very poor, poor, fair, good, very good, and excellent, respectively [4]. Therefore, 1,719 students recruited from 3 separate cohort are assumed to be enough for this study.

### Statistical methods

All data collected were anonymous and the analysis of the data was undertaken by IBM SPSS ver. 26.0 (IBM Corp.) and AMOS ver. 24.0 (IBM Corp.) [5,6]. The statistical analysis consisted of CFA to assess the construct validity of MedSEQ, followed by Cronbach’s α to estimate the factor reliability. Results are reported as root mean square error of approximation (RMSEA), comparative fit index (CFI) and chi-square/degrees of freedom (Cmin/df). Hierarchical linear regressions were used to identify the factors that most impact students’ overall satisfaction with the program. Overall satisfaction was determined by response to the question “All things considered, how do you rate your level of satisfaction with your experience in the UNSW Medicine program?” Possible responses were on a 4-point Likert-scale from very poor to excellent.

## Results

### Participants

The available complete data for this analysis included 1,719 students among 4,983 students in 3 separate cohorts (34.50%), comprising 613 (out of 1,663, 36.86%), 538 (out of 1,658, 32.45%), and 568 (out of 1,662, 34.18%) students in the cohorts of 2017, 2019, and 2021, respectively (Table 1). Regarding location of campus, 1,424 (82.8%), 257 (15%), and 38 (2.2%) students were in metropolitan, rural, overseas, respectively. It was noted that overseas was a campus option only in 2021 due to the impact of COVID-19. In terms of gender, there were 735 (42.8%) men, 976 (56.8%) women, and 8 (0.5%) prefer not to say or other genders.

### Main results

**Fig. 1.**
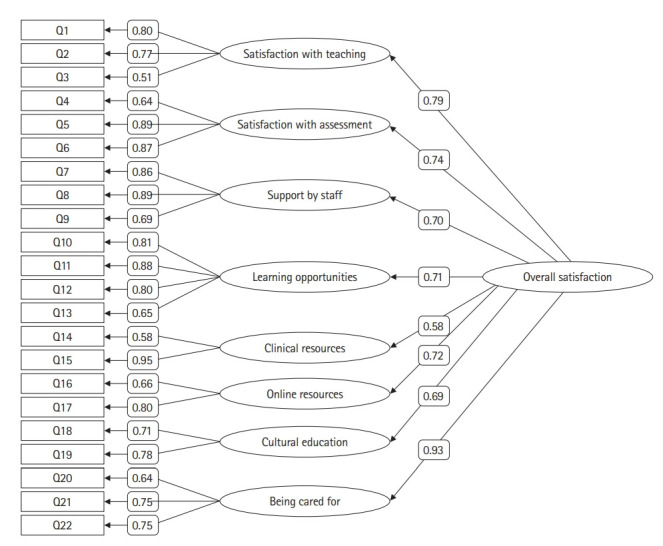
SEM of the Medicine Student Experience Questionnaire (standardized coefficients). SEM, structural equation modelling.

The results of CFA using data from 3 cohorts (Fig. 1) confirm the construct validity of MedSEQ. The overall fit indices were good (RMSEA=0.051; CFI=0.939; Cmin/df=6.429) [7]. Multi-group invariance analysis showed that the measurement weights were not significantly different across the 3 cohorts (P=0.681), indicating that all cohorts shared the same understanding of the MedSEQ questionnaire. A reliability analysis identified that all factors yielded good (α>0.7) or very good (α>0.8) level of reliability, except the “online resources” factor, which yielded an acceptable level of reliability (α=0.687) (Table 2).

The hierarchical multiple linear regression model aimed to identify the factors that most impact students’ overall satisfaction with their experience in the UNSW medicine program. The analysis included all 3 cohorts (2017, 2019, and 2021). Two blocks of predictors were set. The first block included: cohort, current campus of study, gender, and year in the program. The second block included the 8 factors (domains) from the MedSEQ questionnaire (Table 3).

The variables in the first block yielded a model explaining 3.8% of the variance in the level of overall satisfaction and the only statistically significant effect with beta >0.1 (standardized coefficient) was year in the program. Studying in years 3 to 6 had a negative impact on overall satisfaction compared with year 1, whereas studying in year 2 did not significantly change the level of overall satisfaction.

The combined model including 8 domains from MedSEQ explained 40% of variance in students’ overall satisfaction, indicating that 36.2% of the variance is attributable to students’ experience as reported across the 8 domains. In the combined model, year in the program did not have any meaningful impact on overall level of satisfaction (all β<0.1). Only year 4 had a statistically significant negative impact on overall satisfaction, but this effect was negligible (β=-0.06).

Three domains were found to have the strongest impact on students’ overall satisfaction: “being cared for” (β=0.327, P<0.0001); followed by “satisfaction with teaching” and “satisfaction with assessment” (both β=0.148, P<0.0001). All other domains yielded β<0.1. Nonetheless, it is noted that “clinical resources” yielded β=0.081 (P<0.001) and “online learning” yielded β=0.051 (P<0.05), indicating very small impacts, whereas “support by staff,” “learning opportunities,” and “cultural education” had no meaningful impact on overall satisfaction (β<0.02, not significant).

## Discussion

### Key results

MedSEQ demonstrated acceptable validity and reliability, reflecting students’ experiences in the medicine program. Three domains were found to have the greatest impact on student satisfaction: being cared for; satisfaction with teaching; and satisfaction with assessment.

### Interpretation

#### Validity of MedSEQ

MedSEQ reliability and construct validity are supported by the results of this investigation (Fig. 1), which demonstrated that MedSEQ domains yielded acceptable to high reliability (Table 2) and the CFA showed good fit indices (RMSEA=0.051; CFI=0.939; Cmin/df=6.429). It is also noted that MedSEQ construct validity is robust across cohorts, meaning that each cohort of students shared the same understanding of MedSEQ items.

#### Predictors of overall student satisfaction

Understanding the key factors that impact student satisfaction with the medicine program is probably the most important outcome of this study. The results provide interesting insights into these factors (Table 3). Firstly, demographic variables explained only 3.8% of the variance in overall satisfaction, while none of the demographic variables had any meaningful impact. This finding differs from a previous systematic review of 25 studies, which suggested that medical students’ demographic characteristics impact their learning experience [8]. This is an encouraging finding, particularly as it suggests that the UNSW medicine program is equally satisfying for students from diverse backgrounds.

The second important outcome of the regression analysis was that among the 8 domains that might impact student satisfaction, 3 were revealed to be dominant: being cared for, satisfaction with teaching, and satisfaction with assessment. “Being cared for” had the largest impact (β=0.327, P<0.001) which suggests that above and beyond the effectiveness of teaching and clinical resources, students greatly appreciate staff who provide caring learning environments. It is important to emphasize the difference between the support by staff domain, which pertains to administrative support, and being cared for, which relates to the interpersonal interactions between staff and students. Two additional factors found to have significant and meaningful impact on students’ overall satisfaction are satisfaction with teaching and satisfaction with assessment. Twenty years ago, Markert [9] summarized these factors in the following quote: “When colleagues ask me what the most important principles of good teaching are, I say: Be enthusiastic about your teaching and interested in the well-being of your students, prepare well for your teaching, teach knowledge in the context of solving authentic medical problems, and always be thinking about and working on the improvement of your teaching and your students’ learning.” The summary by Markert [9] and insights perfectly apply to our findings.

### Comparison with previous studies

The Association of American Medical Colleges has conducted a Graduation Questionnaire since 1978, that includes more than 25 dimensions including but not limiting to student satisfaction, quality of clerkships, activities in each specialty, learning environment, well-being, scholarship, student indebtedness, etc. [10]. In the 2022 version, most of the questions were related to a specific single experience (i.e., Were you provided with mid-clerkship feedback?), and learning environment and burnout were the only 2 scales with subscales and reliability reported [10]. The former scale has 2 subscales: emotional climate (3 items, Cronbach’s α=0.9) and student-faculty interaction (4 items, α=0.8), whereas the latter has 2 subscales: disengagement (8 items, α=0.8) and exhaustion (8 items, α=0.8). The Graduation Questionnaire has rich data and presents longitudinal changes in each experience and factor. However, most of the experiences were reported by a single question, and no reliability could be identified. In comparison, although MedSEQ presents only 8 factors, the validity was well-established with fair reliability (between 0.687 and 0.856) (Table 2). In addition, MedSEQ demonstrated that 3 factors (satisfaction with teaching, satisfaction with assessment, and being cared for) were most related to student overall satisfaction. In comparison, Graduation Questionnaire did not show related results.

### Limitations

The main limitation of this study is related to the MedSEQ factors, with 3 of them consisting of 2 items each (clinical resources, online resources, and cultural education). This issue was identified in 2017, but for the sake of consistency and to enable the Faculty to make fair longitudinal comparisons of student satisfaction, it was decided to keep these domains of the 2017 MedSEQ version unchanged. It is noteworthy that the CFA demonstrated that factor construct is robust enough and therefore the factors consisting of 2 items did not compromise MedSEQ’s validity. Nonetheless, future improvement of MedSEQ should consider addition of more items relating to the factors comprising only 2 items.

### Generalizability

MedSEQ is valid and reliable to be applied to medical students to reflect their learning experiences. However, relatively low response rates (less than 40%) and non-response bias may potentially undermine the generalizability of the study.

### Suggestions

Future development of the MedSEQ should consider the addition of more items to the 2 item factors.

### Conclusion

MedSEQ has good construct validity and high reliability, reflecting students’ satisfaction with the Medicine program. Medical education has significantly advanced over the past 2 decades, particularly in terms of utilization of advanced technology and online learning. Nonetheless, 3 main core components remain at the center of students’ concerns: (1) they want to feel that they are cared for, (2) to receive quality teaching irrespective of the mode of delivery and (3) to experience fair assessment tasks which enhance their learning. We all need to focus on these domains to further enhance our students’ learning experience and learning outcomes.

## Figures and Tables

**Table 1.** Student distribution by cohort and by study year

	No. of participants (%)	Total no. of participants
MedSEQ cohort		
2017	613 (36.9)	1,663
2019	538 (32.4)	1,658
2021	568 (34.2)	1,662
Year		
Year 1	267 (33.0)	809
Year 2	249 (29.1)	856
Year 3	226 (28.0)	808
Year 4	495 (60.1)	824
Year 5	184 (21.9)	841
Year 6	298 (35.3)	845
Total	1,719 (34.5)	

MedSEQ, Medicine Student Experience Questionnaire.

**Table 2.** Factor reliability (Cronbach’s α)

Domain (factor)	Cronbach’s α
Satisfaction with teaching	0.719
Satisfaction with assessment	0.833
Support by staff	0.850
Learning opportunities	0.856
Clinical resources	0.708
Online resources	0.687
Cultural education	0.712
Being cared for	0.749

**Table 3.** Linear regression: predictors of overall satisfaction with the study

Model	Variable	Category	*B*	*SE B*	β	t-value	Sig.	95% Confidence interval for β
1	(Constant)		3.238	0.047		68.984	0	3.146 to 3.33
	Cohort	2017 (ref)						
		2019	0.032	0.04	0.025	0.784	0.433	-0.048 to 0.111
		2021	0.112	0.035	0.089	3.167	0.002	0.042 to 0.181
	Campus	Metro (ref)						
		Rural	0.079	0.042	0.047	1.872	0.061	-0.004 to 0.161
		Overseas	-0.111	0.101	-0.028	-1.106	0.269	-0.309 to 0.086
	Gender	Male (ref)						
		Female	-0.108	0.034	-0.089	-3.166	0.002	-0.176 to -0.041
		Prefer not to say/other	-0.325	0.223	-0.035	-1.455	0.146	-0.763 to 0.113
	Year	Year 1 (ref)						
		Year 2	-0.009	0.052	-0.005	-0.177	0.86	-0.111 to 0.092
		Year 3	-0.194	0.055	-0.11	-3.544	<0.001	-0.301 to -0.086
		Year 4	-0.204	0.046	-0.156	-4.391	<0.001	-0.295 to -0.113
		Year 5	-0.219	0.058	-0.114	-3.77	<0.001	-0.333 to -0.105
		Year 6	-0.221	0.052	-0.141	-4.215	<0.001	-0.323 to -0.118
2	(Constant)		1.239	0.084		14.666	0	1.074 to 1.405
	Cohort	2017 (ref)						
		2019	-0.036	0.032	-0.028	-1.103	0.27	-0.099 to 0.028
		2021	-0.02	0.03	-0.016	-0.661	0.508	-0.08 to 0.04
	Campus	Metro (ref)						
		Rural	0.095	0.035	0.057	2.717	0.007	0.026 to 0.163
		Overseas	-0.053	0.08	-0.013	-0.667	0.505	-0.211 to 0.104
	Gender	Male (ref)						
		Female	-0.032	0.027	-0.027	-1.182	0.237	-0.086 to 0.021
		Prefer not to say/other	-0.103	0.177	-0.011	-0.583	0.56	-0.451 to 0.245
	Year	Year 1 (ref)						
		Year 2	0.043	0.041	0.026	1.053	0.293	-0.038 to 0.124
		Year 3	-0.039	0.045	-0.022	-0.868	0.386	-0.126 to 0.049
		Year 4	-0.08	0.038	-0.061	-2.118	0.034	-0.155 to -0.006
		Year 5	-0.039	0.048	-0.02	-0.809	0.419	-0.132 to 0.055
		Year 6	0.03	0.044	0.019	0.688	0.491	-0.056 to 0.117
	Satisfaction with teaching		0.117	0.02	0.148	5.886	<0.001	0.078 to 0.155
	Satisfaction with assessment		0.099	0.017	0.148	5.724	<0.001	0.065 to 0.133
	Support by staff		0.013	0.016	0.019	0.765	0.444	-0.02 to 0.045
	Learning opportunities		0.012	0.021	0.014	0.563	0.574	-0.03 to 0.053
	Clinical resources		0.051	0.016	0.081	3.237	0.001	0.02 to 0.083
	Online resources		0.034	0.016	0.051	2.116	0.034	0.003 to 0.066
	Cultural education		0.009	0.015	0.015	0.637	0.524	-0.019 to 0.038
	Being cared for		0.211	0.019	0.327	10.934	<0.001	0.173 to 0.249

Dependent variable: All things considered, how do you rate your level of satisfaction with your experience in the University of New South Wales medicine program? Statistically significant results are marked in bold. Red shading in the Beta column means the values are less than -0.1 or more than 0.1. Yellow shading in Sig. (significance) column means the values are less than 0.05. Ref, reference; B, unstandardized beta; SE B, Standard error for the unstandardized beta; β, standardized beta.

## Footnotes

**Data availability**
Data files are available from Harvard Dataverse: https://doi.org/10.7910/DVN/SQ8PJY Dataset 1. Raw data of response by 3 MedSEQ cohort in 2017, 2019, and 2021.

## Supplementary materials

Supplementary files are available from Harvard Dataverse: https://doi.org/10.7910/DVN/SQ8PJY

Supplement 1. Medicine Student Experience Questionnaire questions items.

Supplement 2. Audio recording of the abstract.
