# Supplementary material for: What impacts students’ satisfaction the most from Medicine Student Experience Questionnaire in Australia: a validity study
Source: J Educ Eval Health Prof. 2023 Jan 18;20:2. doi: 10.3352/jeehp.2023.20.2 (PMC9986309; doi:10.3352/jeehp.2023.20.2)
Supplement: Supplementary file 2 — Supplement 1. Medicine Student Experience Questionnaire questions items. [file jeehp-20-02-suppl.docx]

**Supplement 1.** Medicine Student Experience Questionnaire questions items

All questions below used a 1–6 Likert scale (1=strongly disagree; 6=strongly agree)

| 1. | Teachers/supervisors stimulate my interest in the topics discussed. |
| --- | --- |
| 2. | Teachers/supervisors provide effective feedback that enhances my learning. |
| 3. | Face-to-face learning activities in the medicine program effectively enhance my learning. |
| 4. | I was provided with clear information about key aspects of the medicine program (e.g., graduate capabilities, learning activities, and assessment requirements). |
| 5. | Assessment requirements or expectations in the medicine program are clear to me. |
| 6. | Assessment tasks and requirements are consistent with the learning expectations communicated to me. |
| 7. | Administrative staff on-campus are helpful when I need to resolve academic or personal issues. |
| 8. | Academic staff on-campus are helpful when I need to resolve academic or personal issues. |
| 9. | Administrative staff in clinical settings are helpful when I need to resolve academic or personal issues. |
| 10. | There are adequate opportunities for me to participate in learning communities (e.g., group work and collaborative learning). |
| 11. | There are adequate opportunities for me to improve my teamwork skills. |
| 12. | I have had adequate opportunities (structured and unstructured) to develop my ability to work in diverse teams. |
| 13. | I obtain significant benefits from the learning communities in which I participate. |
| 14. | I have adequate access to patients to support my learning. |
| 15. | The resources I need to support my learning in real clinical settings are readily available (e.g., anatomical models, out-patient clinics, and tutorial rooms). |
| 16. | Online learning activities effectively enhance my learning. |
| 17. | The online learning resources provided by the medicine program are effective in supporting my learning. |
| 18. | The medicine program provided opportunities to reflect on the impact of government policy on the health outcomes of Aboriginal and Torres Strait Islander people. |
| 19. | The medicine program provided opportunities for me to identify, acknowledge and analyze my own cultural values. |
| 20. | My transition between courses and activities within the medicine program have been adequately supported (e.g., pre-clinical to clinical and between phases). |
| 21. | The learning experiences in the medicine program improve my understanding of how medicine is practiced as a profession. |
| 22. | The Faculty cares about my wellbeing. |

**The answers to this question used 4-point Likert scale: 1=very poor; 2=disappointing; 3=good; 4=excellent.**

23. All things considered, how do you rate your level of satisfaction with your experience in the UNSW medicine program?

Domains (items, reliability): satisfaction with teaching (Q1–Q3, α=0.719), satisfaction with assessment (Q4–Q6, α=0.833), support by staff (Q7–Q9, α=0.850), learning opportunities (Q10–Q13, α=0.856), clinical resources (Q14–Q15, α=0.708), online resources (Q16–Q17, α=0.687), cultural education (Q18–Q19, α=0.712), and being cared for (Q20–Q22, α=0.749).
